# Differential antibody responses to gliadin-derived indigestible peptides in patients with schizophrenia

**DOI:** 10.1038/tp.2017.89

**Published:** 2017-05-09

**Authors:** R T McLean, P Wilson, D St Clair, C J Mustard, J Wei

**Affiliations:** 1 Division of Health Research, University of the Highlands and Islands, Centre for Health Science, Inverness, UK; 2 Centre for Rural Health, University of Aberdeen, Centre for Health Science, Inverness, UK; 3 Department of Medicine and Dentistry, University of Aberdeen, Aberdeen, UK

## Abstract

Gluten consumption has previously been implicated in the development of schizophrenia while an immunological link between gluten and schizophrenia was established by the detection of circulating antibodies against gliadin, a major component of wheat gluten. Several studies have reported an increase in circulating antibodies against native gliadin molecules that are unlikely to survive degradation in the digestive system. In this study, therefore, we measured plasma immunoglobulin G (IgG) and IgA antibodies against indigestible gliadin-derived peptide antigens using an in-house enzyme-linked immunosorbent assay (ELISA) among 169 patients with schizophrenia and 236 control subjects. We also examined the plasma levels of IgG and IgA antibodies against the mixture of native gliadins using commercially available ELISA kits. The results showed that patients with schizophrenia had the increased levels of plasma IgG against the γ-gliadin-derived fragment, namely AAQ6C, but decreased levels of plasma IgG against the α- and γ3-gliadin-derived antigens, as compared with control subjects. This study also demonstrated a uniform decrease in plasma IgA antibodies against gliadin-derived antigens. There was no significant difference in the levels of plasma antibodies against native gliadins between the patient group and the control group. Of eight gliadin-derived antigens tested, four showed a sensitivity of >20% against the specificity of ⩾95% for detection of their corresponding antibodies in plasma. These four tests may thus have a potential to serve as biomarkers for the identification of schizophrenia subgroups that may need an alternative therapy or precision treatment. Further investigation with clinical trials should be carried out to explore this possibility.

## Introduction

Schizophrenia is a complex psychiatric disorder, demonstrating heterogeneity in clinical presentation with a combination of positive, negative and cognitive symptoms.^1^ What causes schizophrenia remains unknown but alterations of neuronal communication are believed to underlie the pathophysiology of the illness.^2, 3, 4, 5^ Owing to the diversity of clinical presentation, differences in treatment response and variable epidemiology, it is likely that multifactorial mechanisms contribute to a spectrum of schizophrenic illnesses.^6, 7, 8^

A role of gluten consumption in the development of schizophrenia was initially proposed based on the observation of a positive correlation between national wheat imports and hospital admissions for schizophrenia.^9^ Although the outcomes have been inconsistent, studies have attempted to examine the efficacy of gluten-free diets in the treatment of schizophrenia, demonstrating improvement of clinical scales and earlier recovery in some patients treated with gluten-free diets.^10, 11, 12, 13^ Case studies in the literature have further demonstrated the induction of psychiatric and schizophrenia-like symptoms in response to gluten challenge and the resolution of these symptoms with gluten-free diets.^14, 15^

A mechanism by which gluten consumption may have a role in the development of schizophrenia has yet to be demonstrated. A number of immunological alterations have been found to be associated with schizophrenia, including an increase in pro-inflammatory cytokines and microglia activation.^16, 17, 18, 19^ In addition, an increase in immunoglobulins (Ig) G and A classes against native gliadin, a major component of wheat gluten, was previously observed in a proportion of patients with schizophrenia.^12, 20, 21, 22, 23, 24, 25^ The initiation of Ig production relies upon the recognition and presentation of antigens by the human leukocyte antigen class II (HLA-II) molecules. Genome-wide association studies revealed that the loci most strongly associated with schizophrenia resided in the HLA region.^26, 27, 28^

The epitopes recognized by anti-gliadin antibodies (AGAs) detected in schizophrenia may be different from those identified in the gluten-sensitive enteropathy coeliac disease (CD). Of schizophrenia patients who were positive for AGA IgA, only 3.8% were positive for IgA against CD-specific gliadin-derived epitopes, compared with 12.2% of control subjects.^22, 29^ Furthermore, patients pre-selected for high AGA levels did not display high levels of CD-specific serological markers, such as plasma antibodies against tissue transglutaminase.^30^ Previous studies suggested that the pathogenic gluten fragments for CD were mainly derived from α2-gliadin and γ5-gliadin, whereas immune reactivity to γ3-gliadin and its homologous sequence (NCBI accession AAQ6387) was associated with schizophrenia.^30, 31, 32^

To date, all the tests for circulating AGAs in schizophrenia have been developed with mixtures of full-length native gliadins consisting of ~300 amino acid residues. Such a test would detect antibodies against not only linear epitopes but also conformational epitopes that are unlikely to survive digestion in the gut. In this study, we measured plasma levels of IgG and IgA against indigestible peptide fragments derived from γ- and α-gliadins, which harbour HLA-II restricted epitopes, with an in-house enzyme-linked immunosorbent assay (ELISA) in individuals with schizophrenia and healthy controls. We also tested circulating AGAs in our case–control samples using commercially available ELISA kits.

## Materials and methods

### Subjects

A total of 405 archived plasma samples collected from patients with schizophrenia (*n*=169, 132 males and 37 females, aged 42.0±13.3 years) and control subjects (*n*=236, 159 males and 77 females, aged 44.7±12.5 years), were used to examine the levels of circulating antibodies against gliadin-derived peptide antigens. These samples were collected through the University of Aberdeen in the period between 2003 and 2008, and had been stored long-term at −80 °C without defrosting until they were aliquoted for antibody testing. All patients were diagnosed as having schizophrenia based on the DSM-IV criteria. Control subjects were recruited from a local population in Scotland and screened for psychiatric disorders as described previously.^26^ No case samples were reported to have CD. Both case and control samples were collected in the same period and stored under the same conditions. Antipsychotic drugs used by schizophrenia patients at the time of sampling are listed in Supplementary Table 1, with 128 patients taking a single antipsychotic drug, 14 taking more than one drug and 27 without medication details. All the subjects were classified as British Caucasian and they all gave informed written consent to donate blood samples for research of the pathophysiology of schizophrenia. This study was approved by a local ethics committee and conformed to the provisions of the Declaration of Helsinki.

### Antigen selection

Based on previous literature suggesting immune reactivity against the γ-gliadins in schizophrenia and α-gliadins in CD,^30, 32^ sequences of interest were retrieved from the NCBI protein database (http://www.ncbi.nlm.nih.gov/protein). The sequences were analysed *in silico* to determine indigestible fragments using PeptideCutter software.^33^ The linear peptide antigens used in this study were selected on the basis of the presence of computationally predicted HLA-II binding epitopes.^34, 35^ The resulting sequences were HLA-II restricted and did not contain cutting sites for pepsin, trypsin and chymotrypsin (Table 1). A 29-mer peptide (H-HAQLEGRLHDLPGCPREVQRGFAATLVTN-OH) derived from a maize protein sequence (NCBI accession 1BFA_A) was used as control peptide for nonspecific binding. All peptide antigens were synthesized by solid-phase chemistry with a purity of >95% (Severn Biotech, Worcestershire, UK).

### In-house ELISA for antibodies against gliadin-derived antigens

Each synthetic peptide was dissolved in 67% acetic acid into a 5 mg ml^−1^ stock solution and stored long-term at −20 °C. The working solution was made by diluting the stock solution with phosphate-buffered saline-based coating buffer (P4417, Sigma-Aldrich, Dorset, UK) to 10 μg ml^−1^ for both gliadin-derived antigens and the control antigen; 100 μl working solution was added to each well on Nunc-Immuno Maxisorp 96-well microtiter plates (DIS-971-030 J, Thermo Fisher Scientific, Loughborough, UK). Each plate was coated with two gliadin-derived antigens and the control peptide. After incubation at 4 °C overnight, the plate was washed three times with wash buffer (T9039, Sigma-Aldrich); 100 μl plasma samples were diluted 1:100 in assay buffer (phosphate-buffered saline containing 1.5% bovine serum albumin) for IgA assay and 1:150 for IgG assay, and were added to each sample well. The negative control (NC) wells contained 100 μl assay buffer only. Following incubation for 1.5 h at room temperature, the plate underwent additional washing as described above, and was then incubated for 1.0 h with 100 μl of peroxidase-conjugated goat antibodies either to human IgG (ab98624, Abcam, Cambridge, UK) or to human IgA (A0295, Sigma-Aldrich) diluted 1:30 000–50 000 in assay buffer. The plate underwent additional washing steps; colour development was then initiated by adding 100 μl Stabilized Chromogen (SB02, Life Technologies, Glasgow, UK) and terminated 20 min later with 50 μl Stop Solution (SS04, Life Technologies). The resulting colour change was measured as optical density (OD) at 450 nm with a reference wavelength of 620 nm on a microplate reader. An inter-assay deviation was estimated using quality control samples, which were pooled from 20 to 30 healthy control samples, tested on every 96-well plate, and expressed as a coefficient of variation (CV%) to represent the reproducibility of the in-house ELISA.

Each sample was tested in duplicate. To reduce the interference from nonspecific signals due to the passive absorption of various antibodies in plasma to 96-well microplates, a specific binding index was introduced to express the relative levels of circulating antibodies against gliadin-derived antigen (AGDA). The specific binding index (SBI) was calculated as follows: SBI=[OD_gliadin_−OD_NC_]/[OD_maize_−OD_NC_].

### Testing of AGAs

The plasma AGAs were assayed using commercially available kits for both IgG and IgA against the full-length native gliadin molecules (Omega Diagnostics, Cambridge, UK). All the assays were performed according to the manufacturer’s instructions (http://www.omegadiagnostics.com/). The OD reading of each sample was normalized to the mean OD reading of four-well standards provided for qualitative testing.

### Data analysis

The Kolmogorov–Smirnov test failed to show a normal distribution of AGDA levels in both the patient and control groups (Supplementary Table 2), so the Mann–Whitney *U*-test was applied to examine the differences in AGDA levels and AGA levels between the two groups. Owing to multiple testing, the Bonferroni correction was applied to reduce the type I errors and *P*<0.006 was considered to be statistically significant. The receiver operating characteristic curve analysis was applied to calculate the area under the receiver operating characteristic curve (AUC) with calculation of the ELISA sensitivity against a specificity of ⩾95%. Linear regression was applied to examine which antipsychotic drugs might affect the secretion of circulating AGDA antibodies. In this analysis, the antibody levels were used as a dependent variable, and medication, age and sex were used as the independent variables; Fisher’s combining probability test was applied to determine combined *P*-values based on nine drug-group tests for altered levels of plasma antibodies reacting with each antigen.^36^ Multivariate linear regression was applied to examine the correlations between AGA IgG levels and AGDA IgG levels.

## Results

### Reproducibility of the in-house ELISA

This in-house ELISA had a good reproducibility, in which the inter-assay deviations ranged from 4.6 to 7.5% for AGDA IgA assay and from 9.4 to 16.3% for AGDA IgG assay (Supplementary Table 3).

### Levels of circulating AGDA antibodies

As shown in Table 2, patients with schizophrenia had significantly higher levels of plasma anti-AAQ6C IgG than control subjects (*Z*=−4.65, *P*<0.001), but significantly lower levels of IgG antibodies against AL1G1 (*Z*=−4.65, *P*<0.001) AL2G1 (*Z*=−8.72, *P*<0.001), AL2G2 (*Z*=−6.01, *P*<0.001), ABO3a (*Z*=–6.37, *P*<0.001) and ABO3b (*Z*=−5.32, *P*<0.001). The circulating AGDA IgA levels were all significantly lower in the patient group than the control group (Table 3). Exclusion analysis revealed that the male patients were more likely to contribute to altered AGDA levels in the circulation than the female patients (Supplementary Tables 4 and 5).

### Levels of circulating AGAs

As shown in Table 4, there was no significant difference in plasma AGA IgG levels between the patient group and the control group (*Z*=−0.31, *P*=0.757). Consistent with the direction of previous studies, a nonsignificant increase in plasma AGA IgA levels was observed in patients with schizophrenia (*Z*=−0.22, *P*=0.825).

### Receiver operating characteristic curve analysis

Receiver operating characteristic curve analysis revealed that at a specificity of ⩾95% (Table 5), five assays had a sensitivity of >20%, including anti-AAQ6C IgG assay (20.4%, AUC=0.65), anti-AL2G1 IgG assay (30.7%, AUC=0.76), anti-AL2G2 IgA assay (20.2%, AUC=0.71), anti-ABO3a IgA assay (40.0%, AUC=0.87) and anti-ABOb IgA assay (35.8%, AUC=0.81).

### Effects of antipsychotic medication on antibody secretion

Linear regression analysis demonstrated that quetiapine was the only antipsychotic drug significantly associated with elevated levels of IgG against AAQ6B (adjusted *r*^2^ =0.065, *t*=3.13, *P*=0.002), and eight other antipsychotic drugs did not show a significant association with AGDA IgG levels (Supplementary Table 6); the secretion of AGDA IgA antibodies and AGAs did not appear to be influenced by antipsychotic medication (Supplementary Tables 7 and 8). Fisher’s combining probability test revealed that none of the nine antipsychotic drugs listed in Supplementary Table 1 was significantly associated with the levels of total antibodies against each gliadin-derived antigen in this study (Supplementary Tables 6 and 7).

### Correlation between AGDA antibodies and AGAs

Multivariate linear regression analysis revealed a significant correlation between AGA IgG levels and AGDA IgG levels (Supplementary Tables 9 and 10), in which anti-AL1G1 IgG level was the best predictor of AGA IgG level out of all AGDA IgG antibodies tested in the control group (Standardized *β*=0.20, *P*=0.004), while anti-AAQ6C IgG level was the most significantly correlated to AGA IgG levels in the patient group (Standardized *β*=0.17, *P*=0.037).

## Discussion

This study was undertaken to compare circulating AGDA levels between patients with schizophrenia and healthy controls. The levels of plasma IgG against γ-gliadin-derived antigen AAQ6C were elevated in patients with schizophrenia when compared with healthy controls (Table 2). It is possible that an immune response to the AAQ6C antigen is associated with a subgroup of schizophrenia patients although additional factors, such as their access to the central nervous system, are likely to determine the potential pathological activities of these antibodies in patients with schizophrenia. It has previously been demonstrated that α2-gliadin-derived peptides may not be immunogenic in schizophrenia but are likely to be immunogenic in CD patients.^30^ A genome-wide association study revealed that the DQA1*0501/DQB1*0201 alleles that encode HLA-DQ2.5 molecules conferring a major risk of CD, were significantly less prevalent in schizophrenia cases than healthy controls;^26^ therefore, the decreased levels of circulating antibodies against α-gliadin-derived antigens may partially result from the low frequency of the DQA1*0501/DQB1*0201 alleles in the patient group.

Against all gliadin-derived peptide fragments tested, circulating levels of all AGDA IgA antibodies were significantly lower in schizophrenia patients than healthy controls (Table 3). Although not uniformly observed, a decrease in global IgA levels has been previously measured in patients with schizophrenia and therefore the decrease in AGDA IgA levels may be related to this observation.^37, 38^ The role of gastrointestinal inflammation has recently gained attention in the development of schizophrenia as well as in neurological and psychiatric conditions more generally.^39^ A previous study that examined the markers of gut-inflammation in non-IgE-mediated cow’s milk allergy demonstrated that infants with such an allergy had a significant decrease in serum IgA in response to food challenge accompanied by a decrease in a subclass of IgG specific for α-casein and an increase in gastrointestinal inflammation.^40^ There is also evidence that circulating IgA has an anti-inflammatory role^41^ and decreased IgA levels are commonly found in patients with autoimmune disease.^42^ Accordingly, decreased AGDA IgA levels observed in the present study may reflect dysfunction of immune-regulation and inflammatory processes possibly in the gastrointestinal tract.

Several studies, including a meta-analysis, have suggested an association between increased AGAs for native gliadins and schizophrenia.^12, 20, 21, 22, 23, 25^ However, the present study failed to show a significant increase in either AGA IgG levels or AGA IgA levels, although a nonsignificant increase in AGA IgA levels was observed in patients with schizophrenia (Table 4). All native gliadin molecules consist of ~300 amino acid residues and are unlikely to survive degradation in the digestive system. It is possible that multiple AGAs recognizing distinct epitopes are different between the case group and the control group. Regression analysis examining the correlation between the AGA IgG and the AGDA IgG suggests that anti-AAQ6C IgG is the most predictive of AGA IgG levels in patients with schizophrenia and that anti-AL2G1 IgG is the most predictive of AGA IgG levels in control subjects, suggesting the existence of differential epitopes bound to AGA antibodies in schizophrenia (Supplementary Tables 9 and 10).

Antipsychotic medication is the first-line treatment of schizophrenia but only 50–60% patients show a good response to antipsychotic drugs.^7^ Consequently, there is an urgent need to identify specific biomarkers for precision treatment of the disease. Of eight gliadin-derived antigens tested in this study, four showed a sensitivity of >20% for the detection of their corresponding antibodies in plasma (Table 5). These four tests may thus have a potential to serve as biomarkers for identification of a gluten-related subgroup of schizophrenia, which may be useful for the development of precision treatments.

Owing to the nature of sample collection and the corresponding database information, it was not possible to fully control the potential confounding effects of lifestyle factors, such as alcohol consumption, tobacco use and diets, on the outcomes measured in these case–control samples. Although healthy control subjects were screened for psychiatric illness, there was no additional medical information available and therefore other confounding factors cannot be excluded. Furthermore, the clinical information for patients did not contain consistent reference to clinical subtypes of schizophrenia and so clinical or symptomatic associations for circulating AGDA levels cannot be analysed in this cohort. The lack of antipsychotic-free or drug-naive patients and incomplete medication histories mean that a potential effect of antipsychotic medication on the secretion of anti-gluten antibodies cannot be ruled out, which is a major limitation of this study. Fisher’s combining probability test, however, failed to detect a significant association between antipsychotic medication and circulating anti-gliadin antibody levels (Supplementary Tables 6–8), suggesting that antipsychotic drugs may not significantly affect the secretion of anti-gluten antibodies. Finally, there is an overrepresentation of male subjects in the case group when compared with the control group; the small sample size in females may have underpowered the test for gender differences in antibody secretion.

In summary, this preliminary study demonstrates that altered AGDA levels in the circulation are associated with schizophrenia and could serve as biomarkers for the identification of a schizophrenia subgroup that may need an alternative therapy or precision treatment. Further investigations with clinical trials should be carried out to explore this possibility.

## Figures and Tables

**Table 1:** Sequence information of peptide antigens used for the in-house ELISA

*NCBI accession (antigen)*	*Sequence*	*Position (aa)*	*Native molecule*
CAB76957 (AL1G1)	KCSFQSSQQNPQAQGSVQPQQLPQ	205–226	α1-Gliadin
CAB76964 (AL2G1)	CPFRPQQPYPQSQPQYSQPQQPISQK	88–111	α2-Gliadin
CAB76964 (AL2G2)	KNVYIPPYCTIAPVGIFGTNYR	270–290	α2-Gliadin
AAQ6387 (AAQ6A)	CHFIQPQQPFPQQPQQSFPQQQPSLIK	59–72 110–119	γ-Gliadin
AAQ6387 (AAQ6B)	CHSIIMQQEQQEQRQGVQILVPLSQK	185–208	γ-Gliadin
AAQ6387 (AAQ6C)	HPKCSIMRAPFASIVAGIGGQYRD	253–274	γ-Gliadin
ABO37962 (ABO3a)	KATTIATANMQVDPSGQVQWPQQQPFRC	13–38	γ3-Gliadin
ABO37962 (ABO3b)	KYVRPDCSTINAPFASIVAGISGQH	263–285	γ3-Gliadin

Abbreviation: ELISA, enzyme-linked immunosorbent assay.

Peptide sequences were selected from *in silico* analysis of by PeptideCutter to determine indigestible fragments of >9 amino acid residuals in length, which may have potential antigenicity.

**Table 2:** Levels of circulating IgG against gliadin-derived peptide antigens

*Antigen*	*Control (*n*)*		*Case (*n*)*		Z	P
	*Mean*	±*s.d.*	*Mean*	±*s.d.*		
AL1G1	0.94 (218)	0.20	0.89 (169)	0.18	−4.65	<0.001
AL2G1	1.11 (224)	0.30	0.94 (167)	0.21	−8.72	<0.001
AL2G2	1.28 (224)	0.26	1.19 (167)	0.21	−6.01	<0.001
AAQ6A	1.50 (224)	1.68	1.64 (167)	1.20	−1.19	0.264
AAQ6B	1.16 (222)	0.31	1.36 (167)	0.97	−2.72	0.007
AAQ6C	1.14 (223)	0.29	1.22 (167)	0.26	−4.65	<0.001
ABO3a	1.02 (211)	0.34	0.91 (161)	0.19	−6.37	<0.001
ABO3b	1.01 (211)	0.13	0.95 (161)	0.12	−5.32	<0.001

Mann–Whitney *U*-test was used to test the differences in plasma against gliadin-derived antigen (AGDA) immunoglobulin G (IgG) levels between healthy controls and patients with schizophrenia.

Based on the Bonferroni correction, *P*<0.006 was set as being statistically significant.

**Table 3:** Levels of circulating IgA against gliadin-derived peptide antigens

*Antigen*	*Control (*n*)*		*Case (*n*)*		Z	P
	*Mean*	±*s.d.*	*Mean*	±*s.d.*		
AL1G1	0.83 (222)	0.07	0.81 (166)	0.09	−4.17	<0.001
AL2G1	0.95 (222)	0.11	0.91 (166)	0.11	−7.09	<0.001
AL2G2	1.00 (222)	0.04	0.96 (166)	0.06	−7.20	<0.001
AAQ6A	1.07 (224)	0.21	0.98 (167)	0.23	−7.28	<0.001
AAQ6B	0.93 (224)	0.13	0.90 (167)	0.13	−3.02	0.003
AAQ6C	1.02 (222)	0.08	0.96 (166)	0.08	−6.82	<0.001
ABO3a	1.05 (221)	0.16	0.94 (166)	0.08	−12.51	<0.001
ABO3b	0.92 (221)	0.04	0.87 (166)	0.05	−10.29	<0.001

Mann–Whitney *U*-test was used to test the differences in plasma against gliadin-derived antigen (AGDA) immunoglobulin A (IgA) levels between healthy controls and patients with schizophrenia.

Based on the Bonferroni correction, *P*<0.006 was set as being statistically significant.

**Table 4:** Levels of plasma antibodies against native gliadins

*Antigen*	*Control (*n*)*		*Case (*n*)*		Z	P
	*Mean*	±*s.d.*	*Mean*	±*s.d.*		
AGA IgG	0.64 (226)	0.54	0.65 (168)	0.52	−0.31	0.757
AGA IgA	0.78 (223)	0.49	0.84 (167)	0.72	−0.22	0.825

Mann–Whitney *U*-test was used to compare the differences in plasma anti-gliadin antibody (AGA) levels between healthy controls and patients with schizophrenia. *P*<0.05 was set as being statistically significant.

**Table 5:** ROC curve analysis of plasma anti-gluten antibodies in schizophrenia

*Antibody test*	*Specificity (%)*	*Sensitivity (%)*	*AUC*	*s.e.*	P	*95% CI*
*IgG*						
AAQ6A	95.1	6.0	0.54	0.03	0.243	0.48–0.53
AAQ6B	95.0	8.4	0.58	0.03	0.008	0.52–0.64
AAQ6C	95.1	20.4	0.65	0.03	<0.001	0.59–0.70
AL1G1	95.0	4.2	0.64	0.03	<0.001	0.58–0.70
AL2G1	95.1	30.7	0.76	0.03	<0.001	0.71–0.81
AL2G2	95.1	15.0	0.68	0.03	<0.001	0.63–0.73
ABO3a	95.3	13.8	0.69	0.03	<0.001	0.64–0.75
ABO3b	95.3	7.5	0.66	0.03	<0.001	0.61–0.72
Gliadin	95.1	5.4	0.51	0.03	0.757	0.43–0.55
						
*IgA*						
AAQ6A	95.1	18.0	0.72	0.03	<0.001	0.67–0.77
AAQ6B	95.1	6.6	0.59	0.03	0.002	0.53–0.65
AAQ6C	95.0	16.4	0.70	0.03	<0.001	0.65–0.75
AL1G1	95.0	10.9	0.62	0.03	<0.001	0.57–0.68
AL2G1	95.0	14.5	0.71	0.03	<0.001	0.66–0.76
AL2G2	95.0	20.2	0.71	0.03	<0.001	0.66–0.76
ABO3a	95.0	40.0	0.87	0.02	<0.001	0.83–0.91
ABO3b	95.0	35.8	0.81	0.02	<0.001	0.76–0.85
Gliadin	95.1	6.6	0.49	0.03	0.825	0.44–0.55

Abbreviations: AUC, area under the ROC curve; CI, confidence interval; Ig, immunoglobulin; ROC, receiver operating characteristic.
