# Supplementary material for: Differential antibody responses to gliadin-derived indigestible peptides in patients with schizophrenia
Source: Transl Psychiatry. 2017 May 9;7(5):e1121–. doi: 10.1038/tp.2017.89 (PMC5534957; doi:10.1038/tp.2017.89)
Supplement: Supplementary Information [file tp201789x1.docx]

Supplementary Information

This document contains supplementary data for the manuscript, entitled “Differential Antibody Responses to Gliadin-derived Peptides in Schizophrenia”

# Table S1. Antipsychotic drugs used by schizophrenia patients at the time of sampling

| **Medication** | **Patient (n)** | **Percentage (%)** | **Male (n)** | **Age** (mean ± SD) |
| --- | --- | --- | --- | --- |
| Amisulpride | 9 | 5.3 | 7 | 38.2 ± 15.4 |
| Clozapine | 39 | 22.8 | 30 | 37.4 ± 15.4 |
| Flupenthixol | 28 | 16.4 | 21 | 53.3 ± 9.7 |
| Haloperidol | 6 | 3.5 | 5 | 41.2 ± 11.5 |
| Olanzapine | 30 | 17.5 | 22 | 39.6 ± 11.9 |
| Phenothiazine | 17 | 9.9 | 16 | 45.5 ± 14.6 |
| Quetiapine | 9 | 5.3 | 8 | 48.8 ± 13.8 |
| Risperidone | 14 | 8.2 | 11 | 40.2 ± 12.7 |
| Sulpiride | 4 | 2.3 | 3 | 37.7 ± 22.8 |
| Dual Medication | 14 | 10.4 | 13 | 41.8 ± 8.2 |
| Unknown | 27 | 15.8 | 23 | 50.9±10.8 |

| **Antigen** | **IgG** | | | | **IgA** | | | |
| --- | --- | --- | --- | --- | --- | --- | --- | --- |
|  | **Skewness** | **Kurtosis** | **D Statistic** | **p** | **Skewness** | **Kurtosis** | **D Statistic** | **p** |
| **Case** |  |  |  |  |  |  |  |  |
| AL1G1 | 0.05 | 6.9 | 0.13 | <0.001 | 0.67 | 2.4 | 0.09 | <0.001 |
| AL2G1 | 5.37 | 48.1 | 0.19 | <0.001 | 4.28 | 34.9 | 0.14 | <0.001 |
| AL2G2 | 7.50 | 89.1 | 0.20 | <0.001 | -0.24 | 0.6 | 0.04 | >0.1 |
| AAQ6A | 4.15 | 24.0 | 0.23 | <0.001 | 3.91 | 24.9 | 0.17 | <0.001 |
| AAQ6B | 0.48 | 2.4 | 0.07 | 0.012 | 4.17 | 30.2 | 0.16 | <0.001 |
| AAQ6C | 1.13 | 8.1 | 0.11 | <0.001 | 0.24 | 1.0 | 0.05 | >0.1 |
| ABO3a | 8.91 | 112.4 | 0.20 | <0.001 | 6.42 | 53.0 | 0.25 | <0.001 |
| ABO3b | -1.88 | 9.9 | 0.09 | <0.001 | -0.28 | 1.1 | 0.05 | >0.1 |
| Gliadin | 3.20 | 16.9 | 0.17 | <0.001 | 1.62 | 3.8 | 0.14 | <0.001 |
| **Control** |  |  |  |  |  |  |  |  |
| AL1G1 | 1.31 | 4.9 | 0.09 | 0.001 | 1.88 | 8.3 | 0.13 | <0.001 |
| AL2G1 | 0.83 | 4.4 | 0.13 | <0.001 | 1.63 | 9.9 | 0.12 | <0.001 |
| AL2G2 | 1.11 | 16.8 | 0.15 | <0.001 | -2.32 | 14.8 | 0.12 | <0.001 |
| AAQ6A | 4.40 | 21.9 | 0.29 | <0.001 | 4.45 | 24.7 | 0.26 | <0.001 |
| AAQ6B | 7.14 | 62.0 | 0.28 | <0.001 | 3.90 | 22.4 | 0.19 | <0.001 |
| AAQ6C | 0.59 | 4.6 | 0.10 | <0.001 | 0.02 | 0.6 | 0.07 | 0.048 |
| ABO3a | 0.92 | 1.7 | 0.16 | <0.001 | 2.13 | 10.9 | 0.12 | <0.001 |
| ABO3b | -1.32 | 7.2 | 0.09 | 0.003 | 0.17 | 4.7 | 0.06 | >0.10 |
| Gliadin | 2.08 | 5.8 | 0.16 | <0.001 | 3.37 | 15.1 | 0.18 | <0.001 |

**Table S2. Test for a normal distribution of circulating anti-gluten antibody levels**

Kolmogorov-Smirnov Test was applied to determine a normal distribution of the antibody levels in both the case group and the control group. As the majority of the data has a non-normal distribution, non-parametric statistical tests were applied.

## Table S3. Inter-assay deviation of circulating AGDA levels

| **Antigens** | **Antibody** | **Number of plates** | **CV (%)** |
| --- | --- | --- | --- |
| AL1G1 | IgG | 27 | 15.53 |
|  | IgA | 28 | 5.95 |
| AL2G1 | IgG | 28 | 13.33 |
|  | IgA | 28 | 5.38 |
| AL2G2 | IgG | 28 | 11.02 |
|  | IgA | 28 | 3.23 |
| AAQ6A | IgG | 28 | 16.31 |
|  | IgA | 28 | 6.69 |
| AAQ6B | IgG | 28 | 17.46 |
|  | IgA | 28 | 5.84 |
| AAQ6C | IgG | 28 | 10.53 |
|  | IgA | 28 | 7.57 |
| ABO3a | IgG | 28 | 12.77 |
|  | IgA | 28 | 6.21 |
| ABO3b | IgG | 28 | 9.47 |
|  | IgA | 28 | 4.60 |

The inter-assay deviation was expressed by CV% that was calculated based on the SBI

of the quality control (QC) sample tested on every plate.

## Table S4. Levels of AGDA IgG in plasma samples stratified by gender

| **Antigen** | **Gender** | **Control (n)** | | | **Case (n)** | | | **Z** | **P** |
| --- | --- | --- | --- | --- | --- | --- | --- | --- | --- |
|  |  | **Mean** | **±SD** | **n** | **Mean** | **±SD** | **n** |  |  |
| AL1G1 | Male | 0.96 | 0.22 | 145 | 0.88 | 0.18 | 132 | -4.68 | <0.001 |
|  | Female | 0.91 | 0.15 | 73 | 0.90 | 0.19 | 37 | -1.54 | 0.123 |
| AL2G1 | Male | 1.10 | 0.30 | 152 | 0.94 | 0.21 | 130 | -7.50 | <0.001 |
|  | Female | 1.13 | 0.30 | 73 | 0.94 | 0.22 | 37 | -4.30 | <0.001 |
| AL2G2 | Male | 1.28 | 0.28 | 152 | 1.20 | 0.22 | 130 | -4.66 | <0.001 |
|  | Female | 1.28 | 0.19 | 72 | 1.16 | 0.17 | 37 | -3.82 | <0.001 |
| AAQ6A | Male | 1.55 | 1.36 | 149 | 1.62 | 1.71 | 130 | -1.24 | 0.216 |
|  | Female | 1.38 | 0.81 | 76 | 1.68 | 1.59 | 37 | -0.17 | 0.864 |
| AAQ6B | Male | 1.14 | 0.27 | 148 | 1.42 | 1.08 | 130 | -3.92 | <0.001 |
|  | Female | 1.28 | 0.32 | 74 | 1.14 | 0.30 | 37 | -2.37 | 0.018 |
| AAQ6C | Male | 1.13 | 0.21 | 149 | 1.24 | 0.27 | 130 | -4.89 | <0.001 |
|  | Female | 1.12 | 0.16 | 74 | 1.14 | 0.21 | 37 | -1.08 | 0.279 |
| ABO3a | Male | 1.02 | 0.16 | 144 | 0.91 | 0.20 | 125 | -6.22 | <0.001 |
|  | Female | 1.02 | 0.56 | 67 | 0.93 | 0.17 | 36 | -1.90 | 0.058 |
| ABO3b | Male | 1.02 | 0.12 | 144 | 0.94 | 0.12 | 125 | -5.56 | <0.001 |
|  | Female | 0.98 | 0.16 | 67 | 0.98 | 0.09 | 36 | -0.78 | 0.434 |

Plasma AGDA IgG levels were analysed by gender, with control males compared with case males and control females compared with case females.

## Table S5. Levels of AGDA IgA in plasma samples stratified by gender

| **Antigen** | **Gender** | **Control (n)** | | | **Case (n)** | | | **Z** | **P** |
| --- | --- | --- | --- | --- | --- | --- | --- | --- | --- |
|  |  | **Mean** | **±SD** | **n** | **Mean** | **±SD** | **n** |  |  |
| AL1G1 | Male | 0.83 | 0.08 | 149 | 0.81 | 0.09 | 129 | -3.24 | 0.001 |
|  | Female | 0.83 | 0.06 | 73 | 0.81 | 0.09 | 37 | -2.39 | 0.017 |
| AL2G1 | Male | 0.98 | 0.12 | 149 | 0.91 | 0.11 | 129 | -6.31 | <0.001 |
|  | Female | 0.97 | 0.08 | 73 | 0.93 | 0.09 | 37 | -3.07 | 0.002 |
| AL2G2 | Male | 1.00 | 0.04 | 149 | 0.96 | 0.07 | 129 | -5.75 | <0.001 |
|  | Female | 1.00 | 0.05 | 73 | 0.97 | 0.04 | 37 | -4.19 | <0.001 |
| AAQ6A | Male | 1.07 | 0.23 | 149 | 0.99 | 0.24 | 130 | -5.55 | <0.001 |
|  | Female | 1.05 | 0.17 | 75 | 0.95 | 0.18 | 37 | -4.78 | <0.001 |
| AAQ6B | Male | 0.92 | 0.11 | 149 | 0.90 | 0.12 | 130 | -2.06 | 0.040 |
|  | Female | 0.94 | 0.17 | 75 | 0.90 | 0.14 | 37 | -2.19 | 0.028 |
| AAQ6C | Male | 1.01 | 0.08 | 149 | 0.96 | 0.08 | 129 | -5.18 | <0.001 |
|  | Female | 1.04 | 0.09 | 73 | 0.97 | 0.08 | 37 | -4.09 | <0.001 |
| ABO3a | Male | 1.05 | 0.13 | 149 | 0.94 | 0.07 | 129 | -10.84 | <0.001 |
|  | Female | 1.06 | 0.21 | 72 | 0.95 | 0.09 | 37 | -6.14 | <0.001 |
| ABO3b | Male | 0.92 | 0.03 | 149 | 0.87 | 0.05 | 129 | -8.83 | <0.001 |
|  | Female | 0.92 | 0.04 | 72 | 0.87 | 0.05 | 37 | -5.09 | <0.001 |

Plasma AGDA IgA levels were analysed by gender, with control males compared with case males and control females compared with case females.

## Table S6. **The association between antipsychotic medication and AGDA IgG levels**

| **Drug** | **Regression** | **AAQ6A** | **AAQ6B** | **AAQ6C** | **AL1G1** | **AL2G1** | **AL2G2** | **ABO3a** | **ABO3b** | **Combined p-value** |
| --- | --- | --- | --- | --- | --- | --- | --- | --- | --- | --- |
|  |  |  |  |  |  |  |  |  |  |  |
| Amisulpride | Adj r^2^ | 0.002 | 0.006 | 0.016 | -0.007 | 0.032 | -0.003 | 0.006 | 0.019 | 0.460 |
|  | t | 1.166 | -0.394 | -0.424 | -0.868 | 2.159 | 0.055 | -1.546 | -1.072 |  |
|  | p | 0.246 | 0.694 | 0.672 | 0.387 | 0.032 | 0.956 | 0.124 | 0.286 |  |
| Clozapine | Adj r^2^ | 0.001 | 0.014 | 0.019 | -0.012 | 0.003 | -0.003 | -0.010 | 0.013 | 0.961 |
|  | t | 1.119 | -1.167 | -0.833 | -0.221 | 0.029 | 0.088 | 0.123 | -0.509 |  |
|  | p | 0.265 | 0.245 | 0.406 | 0.825 | 0.977 | 0.930 | 0.903 | 0.612 |  |
| Flupenthixol | Adj r^2^ | -0.002 | 0.008 | 0.02 | -0.012 | 0.003 | 0.007 | -0.008 | 0.015 | 0.997 |
|  | t | 0.914 | -0.594 | -0.884 | 0.186 | 0.205 | -1.252 | 0.534 | 0.713 |  |
|  | p | 0.362 | 0.553 | 0.378 | 0.852 | 0.838 | 0.213 | 0.594 | 0.477 |  |
| Haloperidol | Adj r^2^ | -0.005 | 0.005 | 0.016 | -0.012 | 0.003 | 0.001 | -0.010 | 0.016 | 0.999 |
|  | t | -0.597 | -0.092 | 0.441 | -0.051 | 0.26 | -0.826 | -0.121 | -0.830 |  |
|  | p | 0.551 | 0.927 | 0.660 | 0.960 | 0.795 | 0.410 | 0.904 | 0.408 |  |
| Olanzapine | Adj r^2^ | 0.006 | 0.006 | 0.021 | -0.006 | 0.003 | 0.006 | -0.006 | 0.011 | 0.988 |
|  | t | -1.424 | 0.345 | 1.010 | 1.008 | -0.084 | 1.207 | -0.764 | -0.101 |  |
|  | p | 0.157 | 0.730 | 0.314 | 0.315 | 0.933 | 0.229 | 0.446 | 0.919 |  |
| Phenothiazine | Adj r^2^ | -0.007 | 0.005 | 0.017 | -0.012 | 0.006 | -0.003 | -0.007 | 0.024 | 0.998 |
|  | t | -0.033 | -0.090 | -0.568 | 0.246 | -0.681 | 0.026 | 0.689 | 1.411 |  |
|  | p | 0.974 | 0.928 | 0.571 | 0.806 | 0.497 | 0.979 | 0.492 | 0.16 |  |
| Quetiapine | Adj r^2^ | 0.001 | 0.065 | 0.023 | -0.012 | 0.011 | -0.001 | -0.01 | 0.012 | 0.250 |
|  | t | -1.096 | 3.132 | 1.160 | 0.199 | -1.162 | 0.596 | -0.069 | -0.239 |  |
|  | p | 0.275 | 0.002 | 0.248 | 0.842 | 0.247 | 0.552 | 0.945 | 0.812 |  |
| Risperidone | Adj r^2^ | -0.007 | 0.018 | 0.019 | 0.006 | 0.012 | 0.017 | -0.005 | 0.012 | 0.399 |
|  | t | 0.167 | 1.408 | -0.876 | -1.694 | -1.196 | -1.803 | 0.867 | 0.276 |  |
|  | p | 0.868 | 0.161 | 0.382 | 0.092 | 0.234 | 0.073 | 0.387 | 0.783 |  |
| Sulpiride | Adj r^2^ | -0.002 | 0.019 | 0.043 | -0.011 | 0.012 | 0.035 | -0.008 | 0.016 | 0.218 |
|  | t | -0.901 | -1.467 | -2.135 | 0.367 | -1.206 | -2.498 | -0.591 | -0.827 |  |
|  | p | 0.369 | 0.144 | 0.034 | 0.714 | 0.230 | 0.014 | 0.556 | 0.41 |  |

There was a significant association between quetiapine medication and anti-AAQ6B IgG levels (p=0.002). Based on the Bonferroni correction for individual tests, p<0.006 was set as being statistically significant. All individual p-values were adjusted for ages and genders and the combined p-values were obtained from Fisher’s combined probability test. Adj r^2^: adjusted r^2^

## Table S7. **The association between antipsychotic medication and AGDA IgA levels**

| **Drug** | **Regression** | **AAQ6A** | **AAQ6B** | **AAQ6C** | **AL1G1** | **AL2G1** | **AL2G2** | **ABO3a** | **ABO3b** | **Combined p-value** |
| --- | --- | --- | --- | --- | --- | --- | --- | --- | --- | --- |
|  |  |  |  |  |  |  |  |  |  |  |
| Amisulpride | Adj r^2^ | -0.015 | -0.015 | -0.009 | -0.013 | -0.006 | -0.003 | -0.002 | -0.011 | 0.746 |
|  | t | 0.182 | 0.771 | -0.592 | -0.774 | 0.441 | -0.586 | -1.298 | -0.884 |  |
|  | p | 0.856 | 0.442 | 0.555 | 0.440 | 0.660 | 0.558 | 0.196 | 0.378 |  |
| Clozapine | Adj r^2^ | -0.015 | -0.005 | -0.011 | -0.017 | -0.007 | -0.005 | -0.012 | -0.015 | 0.966 |
|  | t | -0.376 | 1.487 | 0.217 | 0.155 | 0.213 | -0.102 | 0.428 | -0.392 |  |
|  | p | 0.707 | 0.139 | 0.828 | 0.877 | 0.832 | 0.919 | 0.669 | 0.695 |  |
| Flupenthixol | Adj r^2^ | 0.001 | -0.004 | -0.009 | -0.009 | -0.002 | -0.002 | 0.001 | -0.015 | 1.000 |
|  | t | -1.610 | -1.523 | -0.519 | -1.052 | -0.908 | -0.647 | -1.485 | -0.391 |  |
|  | p | 0.109 | 0.130 | 0.604 | 0.294 | 0.365 | 0.518 | 0.140 | 0.696 |  |
| Haloperidol | Adj r^2^ | -0.016 | -0.012 | 0.023 | -0.017 | -0.003 | 0.007 | -0.013 | -0.003 | 0.247 |
|  | t | -0.079 | -1.071 | 2.321 | 0.119 | 0.824 | 1.372 | 0.042 | 1.391 |  |
|  | p | 0.937 | 0.286 | 0.022 | 0.905 | 0.411 | 0.172 | 0.967 | 0.166 |  |
| Olanzapine | Adj r^2^ | -0.016 | -0.019 | -0.010 | -0.011 | 0.002 | 0.000 | -0.009 | -0.012 | 1.000 |
|  | t | -0.009 | -0.034 | -0.414 | 0.935 | 1.219 | 0.870 | 0.768 | 0.744 |  |
|  | p | 0.993 | 0.973 | 0.680 | 0.351 | 0.225 | 0.385 | 0.443 | 0.458 |  |
| Phenothiazine | Adj r^2^ | -0.006 | -0.014 | -0.011 | -0.014 | -0.003 | 0.000 | 0.001 | -0.015 | 1.000 |
|  | t | 1.229 | 0.890 | 0.156 | -0.676 | 0.806 | 0.824 | 1.485 | 0.253 |  |
|  | p | 0.221 | 0.375 | 0.876 | 0.500 | 0.422 | 0.411 | 0.14 | 0.800 |  |
| Quetiapine | Adj r^2^ | 0.006 | -0.016 | -0.011 | -0.011 | -0.007 | -0.005 | -0.011 | -0.016 | 1.000 |
|  | t | 1.853 | -0.663 | 0.209 | 0.911 | 0.097 | -0.093 | 0.633 | 0.064 |  |
|  | p | 0.066 | 0.508 | 0.835 | 0.363 | 0.923 | 0.926 | 0.528 | 0.949 |  |
| Risperidone | Adj r^2^ | -0.014 | -0.014 | -0.010 | -0.016 | 0.008 | -0.001 | -0.013 | -0.014 | 1.000 |
|  | t | 0.478 | 0.888 | 0.362 | -0.244 | -1.540 | -0.800 | -0.046 | 0.432 |  |
|  | p | 0.634 | 0.376 | 0.718 | 0.807 | 0.126 | 0.425 | 0.964 | 0.666 |  |
| Sulpiride | Adj r^2^ | -0.014 | -0.018 | -0.007 | 0.007 | -0.006 | 0.000 | -0.011 | -0.016 | 1.000 |
|  | t | -0.529 | -0.401 | -0.818 | -1.914 | -0.430 | -0.862 | 0.624 | -0.015 |  |
|  | p | 0.598 | 0.689 | 0.415 | 0.057 | 0.668 | 0.390 | 0.534 | 0.988 |  |

There were no significant associations between antipsychotic medication and the levels of IgA against gliadin-derived antigens. Based on the Bonferroni correction for individual tests, p<0.006 was set as being statistically significant. All individual p-values were adjusted for ages and genders and the combined p-values were obtained from Fisher’s combined probability test. Adj r^2^: adjusted r^2^

## Table S8. **The association between antipsychotic medication and AGA levels**

| **Drug** | **Regression** | **AGA IgG** | **AGA IgA** |
| --- | --- | --- | --- |
|  |  |  |  |
| Amisulpride | Adj r^2^ | 0.014 | 0.013 |
|  | t | 0.201 | 2.171 |
|  | p | 0.841 | 0.031 |
| Clozapine | Adj r^2^ | 0.017 | 0.001 |
|  | t | -0.809 | -1.679 |
|  | p | 0.420 | 0.095 |
| Flupenthixol | Adj r^2^ | 0.018 | -0.014 |
|  | t | -0.832 | -0.647 |
|  | p | 0.407 | 0.519 |
| Haloperidol | Adj r^2^ | 0.017 | -0.011 |
|  | t | -0.723 | 0.991 |
|  | p | 0.471 | 0.323 |
| Olanzapine | Adj r^2^ | 0.014 | -0.017 |
|  | t | 0.215 | -0.143 |
|  | p | 0.830 | 0.886 |
| Phenothiazine | Adj r^2^ | 0.017 | -0.015 |
|  | t | -0.755 | 0.517 |
|  | p | 0.452 | 0.606 |
| Quetiapine | Adj r^2^ | 0.018 | -0.016 |
|  | t | -0.866 | 0.357 |
|  | p | 0.388 | 0.721 |
| Risperidone | Adj r^2^ | 0.019 | -0.017 |
|  | t | 0.867 | 0.001 |
|  | p | 0.387 | 0.999 |
| Sulpiride | Adj r^2^ | 0.014 | -0.009 |
|  | t | -0.323 | -1.087 |
|  | p | 0.747 | 0.279 |

Adj r^2^: adjusted r^2^

## Table S9. Multivariate linear regression for the correlation between AGA IgG and

## AGDA IgG in control subjects

| **Antibody** | **Coefficient β** | **Standard Error** | **Standardized Coefficient β** | **p** |
| --- | --- | --- | --- | --- |
|  |  |  |  |  |
| AL1G1 IgG | 0.04 | 0.21 | 0.02 | 0.832 |
| AL2G1 IgG | 0.37 | 0.13 | 0.20 | 0.004 |
| AL2G2 IgG | -0.07 | 0.15 | -0.03 | 0.633 |
| AAQ6A IgG | 0.06 | 0.03 | 0.14 | 0.051 |
| AAQ6B IgG | -0.17 | 0.15 | -0.09 | 0.257 |
| AAQ6C IgG | 0.37 | 0.24 | 0.13 | 0.129 |
| ABO3a IgG | 0.18 | 0.12 | 0.11 | 0.135 |
| ABO3b IgG | -0.31 | 0.34 | -0.08 | 0.363 |

## Table S10. Multivariate linear regression for the correlation between AGA IgG and AGDA IgG in patients with schizophrenia

| **Antibody** | **Coefficient β** | **Standard Error** | **Standardized Coefficient β** | **p** |
| --- | --- | --- | --- | --- |
|  |  |  |  |  |
| AL1G1 IgG | -0.23 | 0.24 | -0.08 | 0.333 |
| AL2G1 IgG | 0.14 | 0.22 | 0.06 | 0.524 |
| AL2G2 IgG | 0.12 | 0.23 | 0.05 | 0.609 |
| AAQ6A IgG | -0.02 | 0.03 | -0.06 | 0.474 |
| AAQ6B IgG | 0.00 | 0.05 | 0.00 | 0.963 |
| AAQ6C IgG | 0.34 | 0.16 | 0.17 | 0.037 |
| ABO3a IgG | 0.39 | 0.30 | 0.14 | 0.202 |
| ABO3b IgG | -0.80 | 0.49 | -0.18 | 0.103 |

| **Antibody** | **Coefficient β** | **Standard Error** | **Standardized Coefficient β** | **p** |
| --- | --- | --- | --- | --- |
|  |  |  |  |  |
| AL1G1 IgG | 1.69 | 0.49 | 0.25 | 0.001 |
| AL2G1 IgG | 0.06 | 0.32 | 0.01 | 0.851 |
| AL2G2 IgG | -0.08 | 0.87 | -0.01 | 0.925 |
| AAQ6A IgG | 0.14 | 0.16 | 0.06 | 0.394 |
| AAQ6B IgG | -0.28 | 0.33 | -0.06 | 0.385 |
| AAQ6C IgG | -0.46 | 0.43 | -0.08 | 0.287 |
| ABO3a IgG | 0.39 | 0.21 | 0.13 | 0.060 |
| ABO3b IgG | -0.43 | 1.09 | -0.03 | 0.693 |

## Table S11. Multivariate linear regression for the correlation between AGA IgA and AGDA IgA in control subjects

| **Antibody** | **Coefficient β** | **Standard Error** | **Standardized Coefficient β** | **p** |
| --- | --- | --- | --- | --- |
|  |  |  |  |  |
| AL1G1 IgG | 0.70 | 0.71 | 0.09 | 0.328 |
| AL2G1 IgG | 0.36 | 0.81 | 0.05 | 0.661 |
| AL2G2 IgG | -1.29 | 1.12 | -0.11 | 0.249 |
| AAQ6A IgG | -0.45 | 0.37 | -0.14 | 0.221 |
| AAQ6B IgG | 0.22 | 0.65 | 0.03 | 0.742 |
| AAQ6C IgG | 0.03 | 0.81 | 0.01 | 0.973 |
| ABO3a IgG | 2.20 | 0.93 | 0.23 | 0.019 |
| ABO3b IgG | -1.49 | 1.47 | -0.10 | 0.313 |

## Table S12. Multivariate linear regression for the correlation between AGA IgA and AGDA IgA levels in patients with schizophrenia

**A**

**B**

**C**

**F**

**E**

**D**

**G**

**H**

**Figure S1. Scatter plots of AGDA IgG levels in case-control samples**

**A:** AL1G1, **B:** AL2G1, **C:** AL2G2, **D:** AAQ6A, **E:** AAQ6B, **F:** AAQ6C, **G:** ABO3a, and **H:** ABO3b

**A**

**B**

**C**

**F**

**E**

**D**

**G**

**H**

**Figure S2. Scatter plots of AGDA IgA levels in case-control samples**

**A:** AL1G1, **B:** AL2G1, **C:** AL2G2, **D:** AAQ6A, **E:** AAQ6B, **F:** AAQ6C, **G:** ABO3a, and **H:** ABO3b
